# Altered Behavior in Mice with Deletion of the Alpha2-Antiplasmin Gene

**DOI:** 10.1371/journal.pone.0097947

**Published:** 2014-05-29

**Authors:** Eri Kawashita, Yosuke Kanno, Kanako Ikeda, Hiromi Kuretake, Osamu Matsuo, Hiroyuki Matsuno

**Affiliations:** 1 Department of Clinical Pathological Biochemistry, Faculty of Pharmaceutical Science, Doshisha Women’s College of Liberal Arts, Kyo-tanabe, Kyoto, Japan; 2 Department of Physiology II. Kinki University School of Medicine, Osakasayama, Osaka, Japan; Chiba University Center for Forensic Mental Health, Japan

## Abstract

**Background:**

The α2-antiplasmin (α2AP) protein is known to be a principal physiological inhibitor of plasmin, and is expressed in various part of the brain, including the hippocampus, cortex, hypothalamus and cerebellum, thus suggesting a potential role for α2AP in brain functions. However, the involvement of α2AP in brain functions is currently unclear.

**Objectives:**

The goal of this study was to investigate the effects of the deletion of the α2AP gene on the behavior of mice.

**Methods:**

The motor function was examined by the wire hang test and rotarod test. To evaluate the cognitive function, a repeated rotarod test, Y-maze test, Morris water maze test, passive or shuttle avoidance test and fear conditioning test were performed. An open field test, dark/light transition test or tail suspension test was performed to determine the involvement of α2AP in anxiety or depression-like behavior.

**Results and Conclusions:**

The α2AP knockout (α2AP^−/−^) mice exhibited impaired motor function compared with α2AP^+/+^ mice. The α2AP^−/−^ mice also exhibited impairments in motor learning, working memory, spatial memory and fear conditioning memory. Furthermore, the deletion of α2AP induced anxiety-like behavior, and caused an anti-depression-like effect in tail suspension. Therefore, our findings suggest that α2AP is a crucial mediator of motor function, cognitive function, anxiety-like behavior and depression-like behavior, providing new insights into the role of α2AP in the brain functions.

## Introduction

α2-Antiplasmin (α2AP), a member of the serine protease inhibitor (serpin) family, is a glycoprotein with a molecular weight of approximately 70 kDa, and is a principal physiological plasmin inhibitor [1]–[5]. Lysines at the C-terminus of α2AP bind to lysine-binding sites in the kringle domains of plasmin and its precursor, plasminogen (Plg), thus regulating in fibrinolysis and proteolysis. α2AP also regulates myofibroblast differentiation and neuronal morphology, independent of plasmin [6]–[9].

Many studies have reported that the extracellular proteolysis by plasmin or tissue plasminogen activator (tPA) regulates the synaptic plasticity, cognitive function and anxiety [4], [10]–[16]. The deletion of the tPA gene of mice or the treatment with tPA inhibitor in the hippocampus of mice shows an interference with late-phase long-term potentiation (L-LTP) [4], [10]. The neuronal expression of plasminogen or tPA is involved in hippocampus-dependent learning or stress-induced response, including cognitive decline, depression- and anxiety-like behaviors [11]–[16]. On the other hand, our previous study demonstrated that α2AP induces filopodia formation, dendritic elongation and branching in hippocampal neurons, independent of its effects on plasmin [9]. α2AP is mainly produced by the liver and kidneys; however, it is also expressed in various regions in the brain, including the hippocampus, cortex and cerebellum [17]–[18]. These findings suggest that α2AP might play important roles in brain functions in both a plasmin-dependent and plasmin-independent manner. However, the role of α2AP in the brain has not been sufficiently addressed. In this study, we demonstrate that α2AP may be a crucial regulator of motor function, cognitive function, anxiety-like and depression-like behavior.

## Results and Discussion

### Impairments in Motor Function and Motor Learning in α2ap^−/−^ Mice Compared with WT Mice

**Figure 1:**
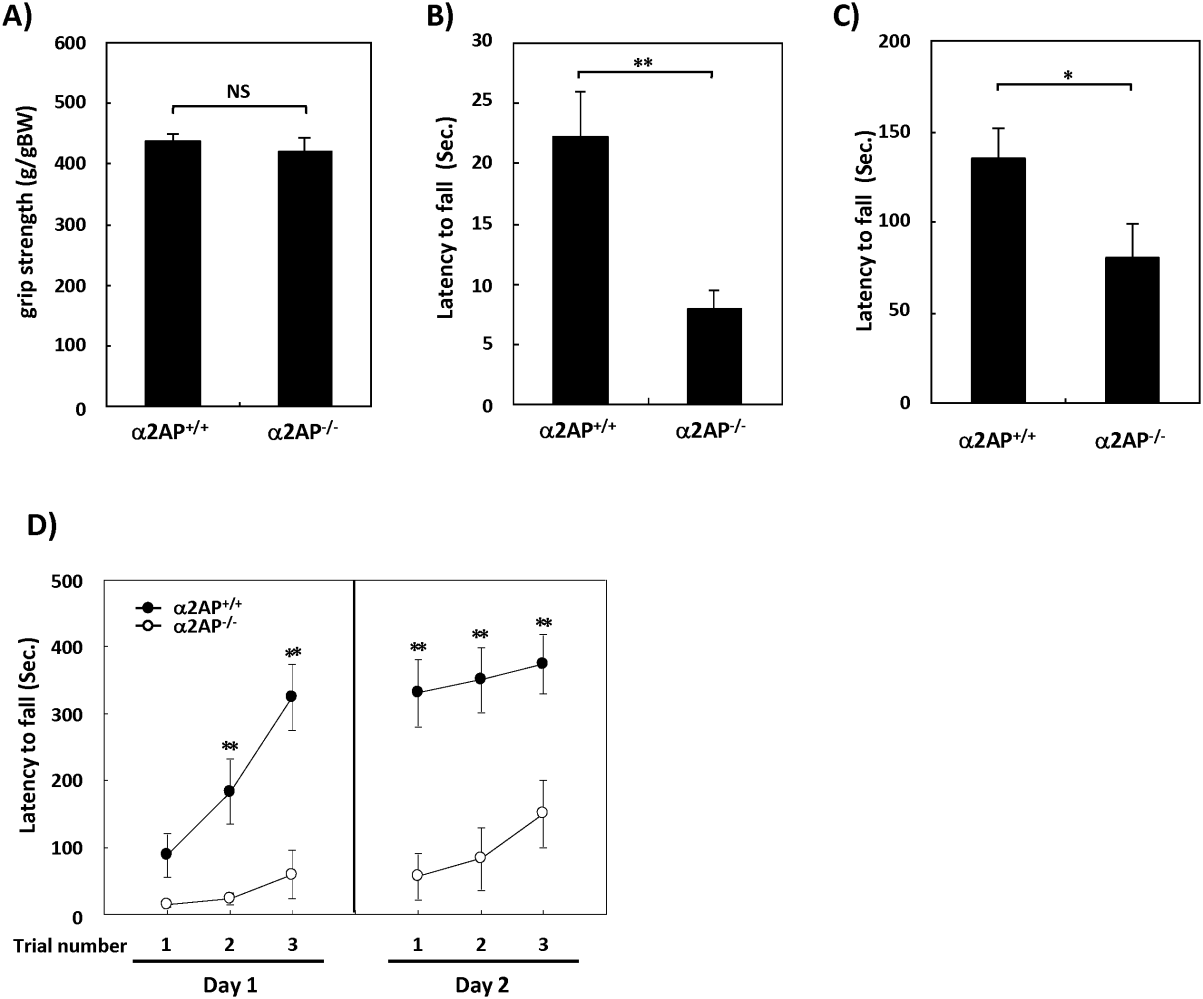
The impaired motor function in α2AP^−/−^ mice compared with WT mice. The grip strength was measured by the traction test (A). There were no differences between the α2AP^−/−^ and α2AP^+/+^ mice (WT and α2AP^−/−^ mice, n = 26 and 15, respectively). The wire hang test using a cage top (B) and wire hang test using a wire (C) showed impaired motor function in α2AP^−/−^ mice compared with WT mice (WT and α2AP^−/−^ mice, n = 14 and 10, respectively). The rotarod test showed that the α2AP^−/−^ mice exhibited impaired motor function and learning (WT and α2AP^−/−^ mice, n = 16 and 14, respectively) (D). The values represent the means±S.E. Significance was evaluated using Student’s *t*-test or an ANOVA with a LSD post-hoc test. *P<0.05, **P<0.01.

The α2AP^−/−^ mice exhibited no evident abnormalities in a neurological screening, including the righting reflex, whisker twitch, ear twitch, blink reflex and postural reflex. The traction test and the wire hang test are both used to measure muscle strength, but the wire hang test evaluates not only the muscle strength, but also the coordination and balance. The traction test showed no significant differences in the grip strength between the two types of mice (Fig. 1A), while the wire hang test using the cage top or a wire indicated that the latency to fall was significantly shorter in α2AP^−/−^ mice than in WT mice (Figs. 1B and C). Moreover, in the first trial of the rotarod test for motor coordination and balance, the latency to fall tended to be shorter in α2AP^−/−^ mice compared with WT mice (Fig. 1D). These results suggest that the deletion of the α2AP gene causes impaired motor function, but no major differences in muscle strength.

The rotarod test is also used to evaluate motor learning by repeating trials. Both types of mice exhibited increased latencies to fall, but the latency of the α2AP^−/−^ mice was obviously shorter than that of the WT mice, suggesting that the deletion of the α2AP gene also causes impaired motor learning.

### Impaired Cognitive Function in α2ap^−/−^ Mice Compared with WT Mice

**Figure 2:**
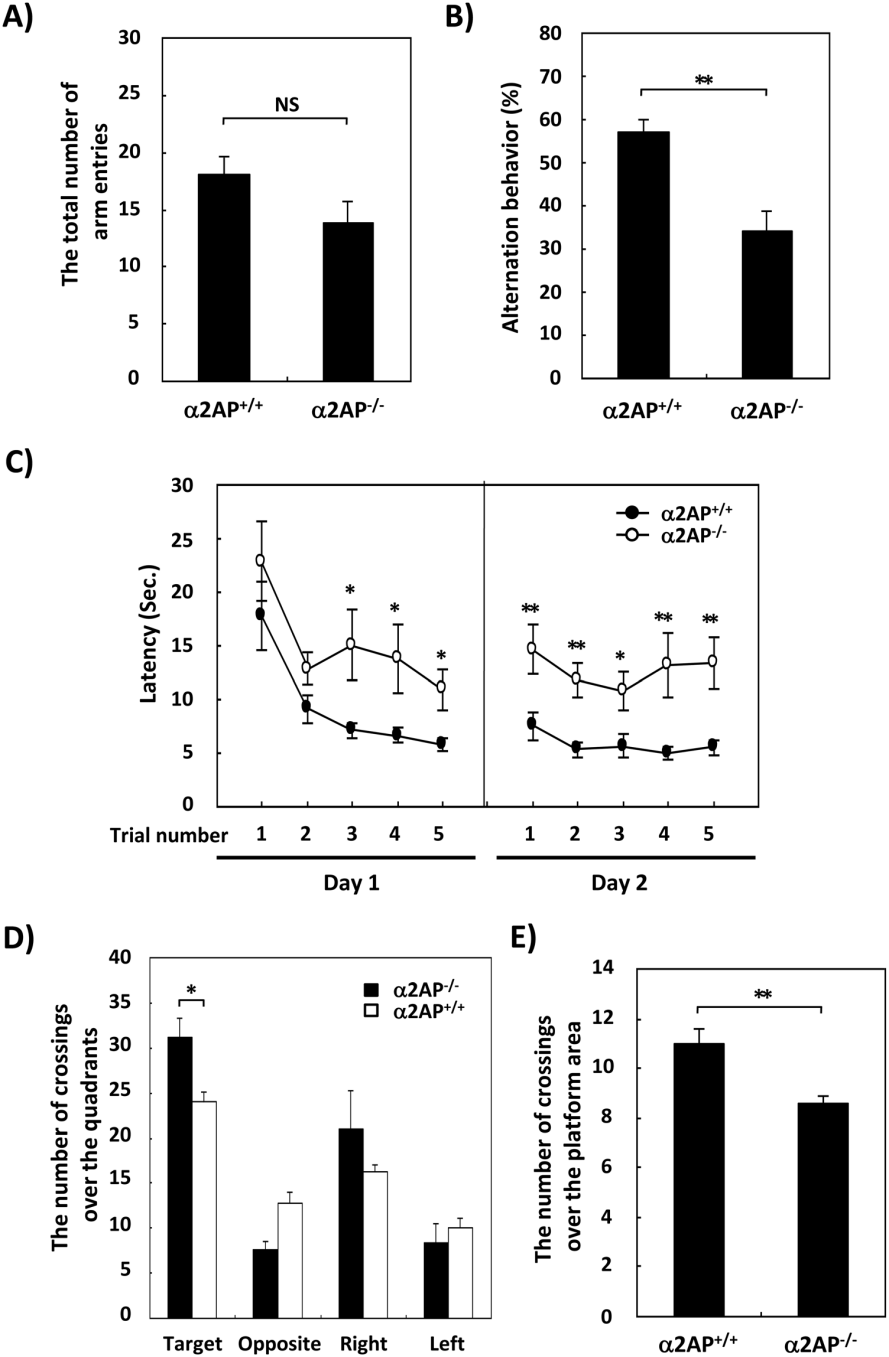
The impaired working memory and spatial memory in α2AP^−/−^ mice compared with WT mice. The Y-maze test showed that there was little effect of the α2AP deficiency on the spontaneous behavior of the mice (A), but the working memory was impaired in the α2AP^−/−^ mice compared with WT mice (B) (WT and α2AP^−/−^ mice, n = 12 and 8, respectively). The results of the training sessions are shown in C. The number of crossings in each quadrant and the number of crossings over the platform in the probe test are shown in D and E, respectively. The Morris water maze test showed that α2AP^−/−^ mice exhibited impairments in their spatial memory (C–E) (n = 8). The values represent the means±S.E. Significance was evaluated using Student’s *t*-test or an ANOVA with a LSD post-hoc test. *P<0.05, **P<0.01.

To determine the effects of α2AP deficiency on the cognitive function, the Y-maze test and Morris water maze (MWM) test were performed. In the Y-maze test, there were minimal differences in the total numbers of arm entries, which indicates the amount of spontaneous behavior, between the α2AP^−/−^ and WT mice (Fig. 2A). However, the alteration behavior, indicating the working memory, was significantly lower in the α2AP^−/−^ mice compared with the WT mice (Fig. 2B). In the training sessions of the MWM test, the latency to reach the platform gradually decreased in both types of mice by the repeated training sessions, but the latency of the α2AP^−/−^ mice was a couple of fold longer than that of the WT mice (Fig. 2C). The swimming speeds of the WT and α2AP^−/−^ mice were 17.7±0.4 and 15.4±0.7 cm/sec, respectively. However, the subtle difference in the swimming speeds could not account for the more than 2 fold difference in the escape latency between the WT and α2AP^−/−^ mice. Furthermore, in the probe test, the number of crossings in the quadrant where the platform had been and the number of crossings over the platform area were significantly lower in the α2AP^−/−^ mice than in the WT mice (Figs. 2D and E, respectively). These results indicate that the deletion of the α2AP gene also caused impaired cognitive function.

**Figure 3:**
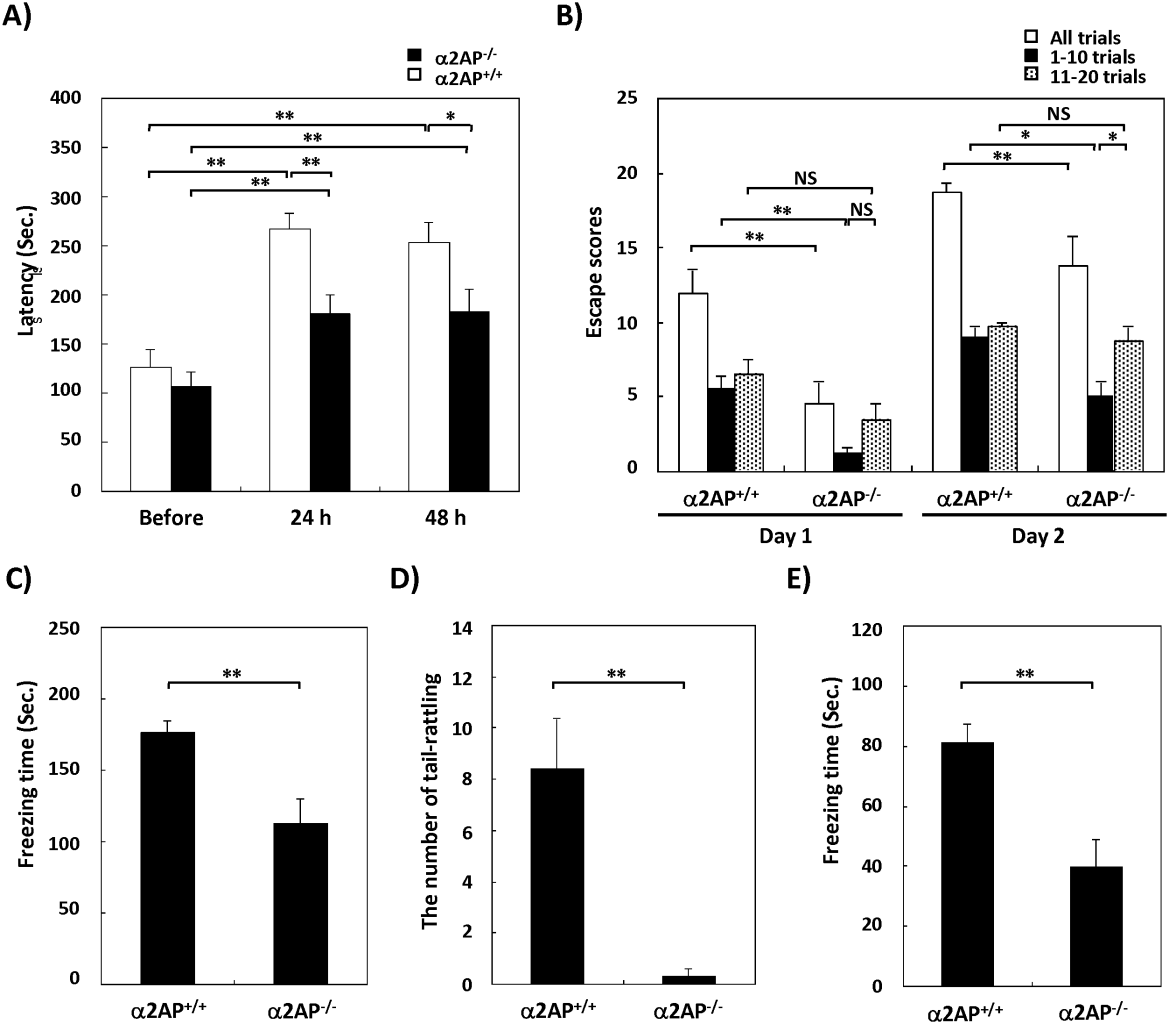
The impaired affect memory in α2AP^−/−^ mice compared with WT mice. The passive avoidance test (A) (WT and α2AP^−/−^ mice, n = 24 and 31, respectively), and shuttle avoidance test (B) (n = 4 for both groups) showed that the deletion of α2AP results in impaired affect memory. Furthermore, α2AP^−/−^ mice exhibited impaired memory in the contextual fear conditioning test (C and D), and the cued fear conditioning task (E) (WT and α2AP^−/−^ mice, n = 14 and 10, respectively). The values represent the means±S.E. Significance was evaluated using Student’s *t*-test or an ANOVA with a LSD post-hoc test. *P<0.05, **P<0.01.

Next, we assessed the effects of α2AP deficiency on affect memory. We confirmed that there was no significant difference in the pain sensitivity between α2AP^−/−^ and WT mice by the hot plate test (Figure S1). In the passive avoidance test, the latency to enter the dark compartment was significantly shorter in α2AP^−/−^ mice than that of WT mice after the electric shock (Fig. 3A). A similar result was found in the shuttle avoidance test. The total escape scores of the α2AP^−/−^ mice were lower than those of the WT mice on both days (Fig. 3B). Interestingly, the escape scores in the first half of the test were significantly lower in the α2AP^−/−^ mice compared with the WT mice, but in the second half of the test, there was no significant difference between the types of mice, suggesting that the deletion of the α2AP causes a cognitive delay. Moreover, in the contextual fear conditioning task, the freezing time and the number of occurrences of tail-rattling were remarkably lower in the α2AP^−/−^ mice compared with the WT mice (Figs. 3C and D). In addition, in the cued fear conditioning task, the freezing time of the α2AP^−/−^ mice was significantly lower than that of the WT mice (Fig. 3E). We also confirmed that α2AP^−/−^ mice have an intact electric shock-induced acute freezing response (Figure S2). These results suggest that α2AP plays an important role in affect memory, and possibly in the etiology of posttraumatic stress disorder (PTSD) related to the fear-based memory formation [20].

### The Effects of the Deletion of the α2ap Gene on Anxiety-like or Depression-like Behavior

**Figure 4:**
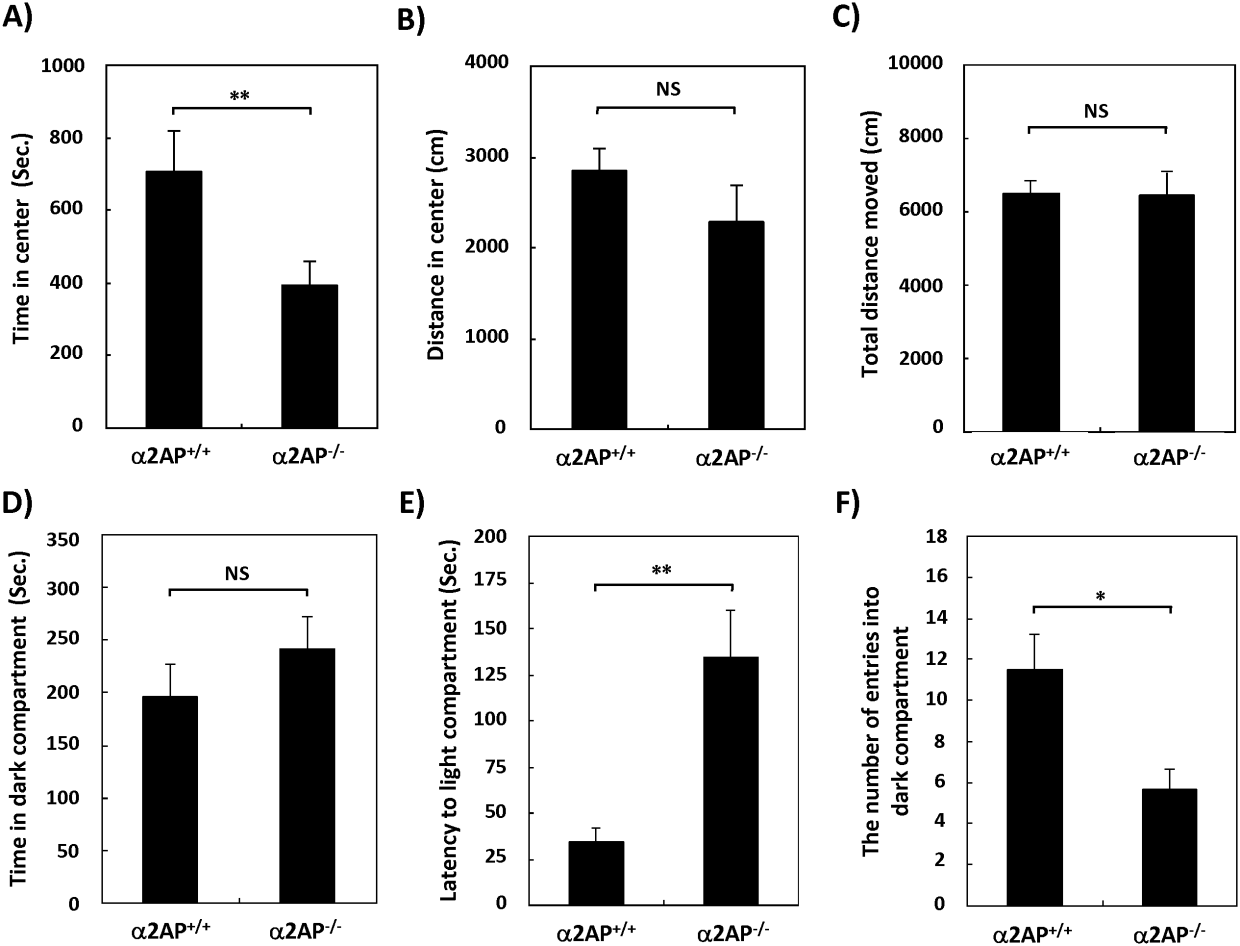
The effects of α2AP deficiency on anxiety-like behavior. The open field test showed that α2AP^−/−^ mice exhibited a shorter time in the center, indicating anxiety-like behavior (A), while there was little difference in the distance moved (B and C) (WT and α2AP^−/−^ mice, n = 11 and 10, respectively). In the dark/light transition test, there was little difference in the time spent in the dark compartment (D). However, the first latency to enter the light compartment was longer (E), and the number of transitions in the α2AP^−/−^ mice was lower than in the WT mice (F), indicating that the deletion of α2AP caused anxiety-like behavior (WT and α2AP^−/−^ mice, n = 11 and 10, respectively). The values represent the means±S.E. Significance was evaluated using Student’s *t*-test or an ANOVA with a LSD post-hoc test. *P<0.05, **P<0.01.

To determine the effects of α2AP deficiency on anxiety-like behavior, the open field test and dark/light transition test were performed in α2AP^−/−^ and WT mice. In the open filed test, the time spent in the center of the field was significantly shorter in the α2AP^−/−^ mice than in the WT mice (Fig. 4A), while there was no significant differences in the distance in the center nor the total distance moved in the field (Figs. 4B and C). In the dark/light transition test, there was little difference in the time spent in the dark compartment (Fig. 4D), but the first latency for the α2AP^−/−^ mice to move to the light compartment was remarkably longer than that of the WT mice (Fig. 4E). In addition, the number of transitions was significantly lower in the α2AP^−/−^ than in the WT mice (Fig. 4F). These results suggest that the deletion of α2AP induces anxiety-like behavior.

**Figure 5:**
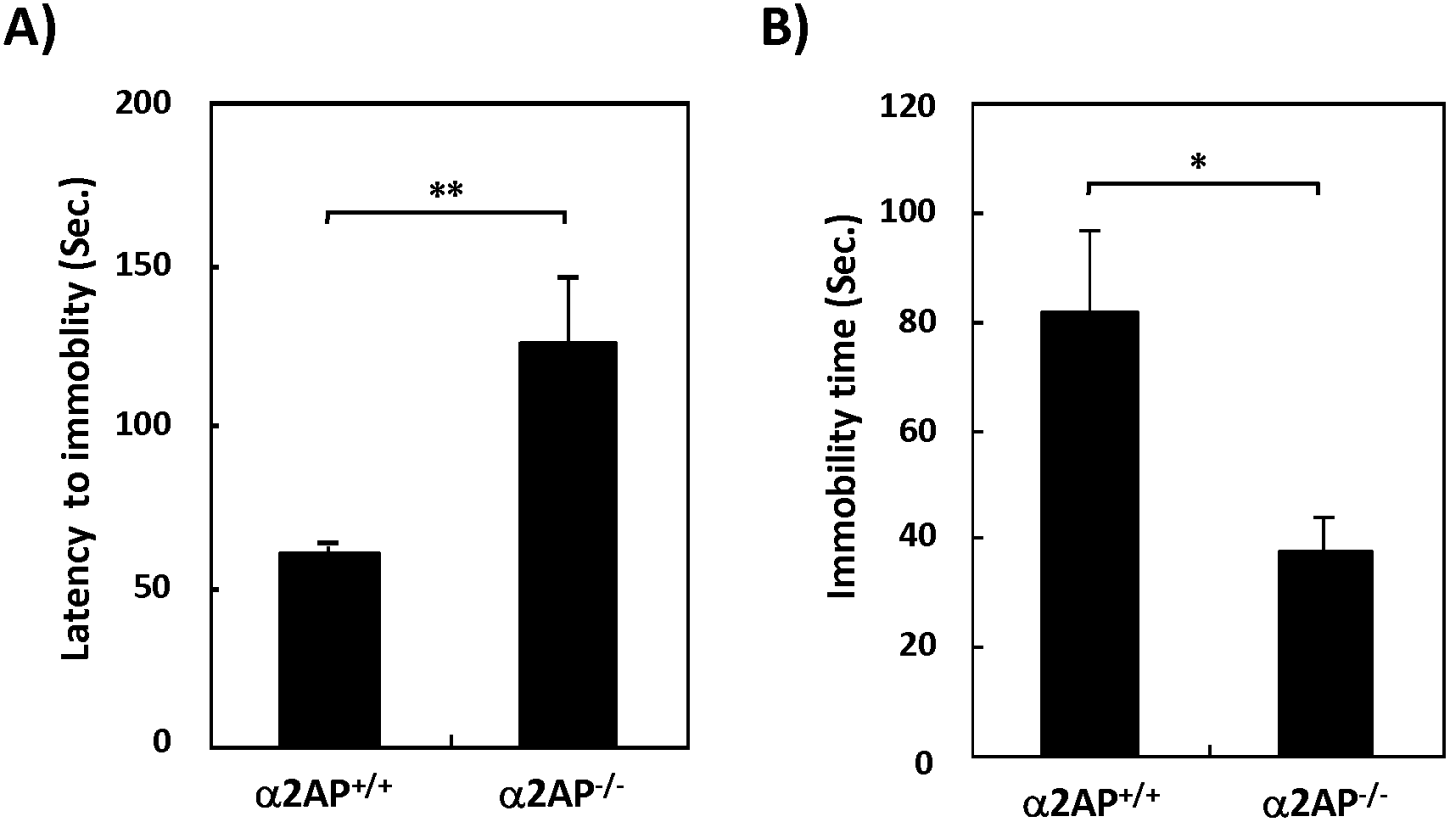
The effects of α2AP deficiency on depression-like behavior. The tail suspension test showed that α2AP^−/−^ mice exhibited an anti-depression-like reaction (A and B) (WT and α2AP^−/−^ mice, n = 14 and 9, respectively). The values represent the means±S.E. Significance was evaluated using Student’s *t*-test or an ANOVA with a LSD post-hoc test. *P<0.05, **P<0.01.

Next, to examine the involvement of α2AP in depression-like behavior, the tail suspension test was performed. The α2AP^−/−^ mice showed a longer first latency to immobility and a shorter immobility time compared with WT mice (Figs. 5A and B), indicating that α2AP^−/−^ mice exhibit anti-depression-like reaction.

The tPA/plasmin proteolytic cascade is known for its thrombolytic ability [21], while the extracellular proteolysis involved in the tPA/plasmin cascade has been reported to extend to synaptic plasticity in the CNS. The cleavage of brain-derived neurotrophic factor (BDNF) by tPA/plasmin cascade is critical for the production of L-LTP in the hippocampus [4]. However, the laminin degradation induced by plasmin results in the impairment of LTP in the hippocampus [22]. Plasmin also disrupts mossy fiber axon guidance [23], and excess tPA/plasmin suppresses dendritogenesis and synaptogenesis [24]. These reports imply that the tPA/plasmin cascade affects synaptic plasticity both positively and negatively. The proteolysis induced by plasmin potentiates N-methyl-D-aspartate receptor responses [25], [26], which may be involved in the enhancement of synaptic plasticity and/or neuronal toxicity. The degradation of the extracellular matrix activated by the tPA/plasmin cascade upregulates the motility of dendritic spine [27], and induces neuronal detachment and apoptosis [28]. These molecular mechanisms may underlie the effects of tPA/plasmin on neuronal remodeling. α2AP is widely known to be a principal physiological inhibitor of plasmin in the thrombolytic system [1], and is expressed in various regions in the brain, including the hippocampus, cortex and cerebellum [18]. However, only a few studies have focused on the role of α2AP in the CNS. One study demonstrated that chronic injection of α2AP into the medial prefrontal cortex inhibits the NGF maturation induced by plasmin, causing cholinergic degeneration and cognitive impairment [29]. On the other hand, we previously demonstrated that α2AP induces dendritic elongation and branching, which are essential for synaptic plasticity and memory formation, independent of plasmin [9]. Therefore, fibrinolytic factors, including tPA, plasmin and α2AP, regulate synaptic plasticity, both positively and negatively, in accordance with physiological and pathological conditions.

In this study, we demonstrated that deletion of the α2AP gene results in an impaired cognitive function. Such failure in the regulation of plasmin activity in the brain and/or the loss of α2AP-regulating neuronal outgrowth may lead to impaired synaptic plasticity in α2AP^−/−^ mice. In addition, recent studies have demonstrated that tPA/plasmin plays a role in the disruption of the blood-brain barrier [30], [31], and that plasminogen potentiates thrombin neurotoxicity in cases of intracerebral hemorrhage [32]. The increase in the permeability of the blood-brain barrier induced by plasmin is possibly involved in the impaired cognitive function observed in α2AP^−/−^ mice. A previous clinical study showed that the plasma levels of α2AP are lower in elderly people [33], suggesting that α2AP may play a role in the age-related cognitive decline. We also demonstrated that α2AP is involved in the development of anxiety- and depression-like behaviors. Deletion of the tPA gene affects anxiety-like behavior [14], [16], although it has not been sufficiently addressed whether plasmin plays a role in this effect. BDNF, which is converted to the mature form by extracellular proteases, including plasmin [34], has an antidepressant-like effect [35], [36]. Hence, free plasmin may mediate the anti-depression-like reactions noted in α2AP^−/−^ mice.

In summary, we herein demonstrated, for the first time, that α2AP is a crucial mediator of the motor and cognitive functions as well as anxiety- and depression-like behaviors. tPA, plasmin and α2AP are each involved in the processes of neuronal migration, dendritic growth and synaptic plasticity [4], [9]–[16], [21]–[29], [37], suggesting that α2AP has an effect on both brain development and neuronal plasticity during behavior. Although further research is needed to elucidate the timing at which α2AP regulates neuronal functions and the molecular mechanisms underlying the regulatory processes controlled by α2AP, our findings provide new insight into the physiological and pathological roles of α2AP in the brain.

## Materials and Methods

### Animals

The α2AP-deficient (α2AP^−/−^) mice were generated by homologous recombination using 129/SvJ RW4 embryonic stem cells, as described previously [19]. To minimize the variability in the genetic background of the mice, we repeatedly backcrossed α2AP^−/−^ mice to C57BL/6J mice for more than 10 generations (≥99.9% of C57BL/6J genomic background). The α2AP^−/−^ and control α2AP^+/+^ (wild-type, WT) mice used for behavioral tests were homozygously bred. All experiments were approved by the institutional animal care and use committee of Doshisha Women’s College (Permit number: Y13-022), and were performed in accordance with the institutional guidelines. All efforts were made to minimize suffering.

Experimentally-naive mice were used for the Morris water maze test, rotarod test, passive avoidance test and shuttle avoidance test. The other behavioral tests were performed with the same group of mice in accordance with the behavioral test battery. The order of the behavioral tests was as follows: open field test, dark/light transition test, wire hang test, traction test, hot plate test, Y-maze test, fear conditioning test and tail suspension test.

### Traction Test

The grip strength of the mice was measured with a traction meter (BrainScience idea.Co., Ltd., Osaka, Japan). Mice were made to grasp metal mesh with all four limbs, and were slowly pulled back using the tail. The maximum tension was recorded and normalized to the body weight.

### Wire Hang Test

Mice were placed on a cage top or a wire. The cage top was slightly shaken to encourage gripping of the bars, and then was slowly inverted. The latency to fall was then measured up to 60 sec.

### Rotarod Test

The rotarod test was performed using a Rota-Rod Treadmill (Muromachi Kikai, Tokyo, Japan). Mice were made to walk for a maximum of 500 sec. The time it took for a mouse to lose its balance on the rod was measured. Mice received three trials at 20 min intervals per day, and the trials were repeated on the next day.

### Y-maze Test

The Y-maze apparatus consisted of three arms whose walls had different markings. Mice were placed in the center and allowed to explore the apparatus for 8 min, while being monitored by a video-tracking system (SMART, Panlab, Barcelona, Spain). The alteration behavior was calculated as the ratio of the number of alterations to the total number of arm entries minus 2.

### Morris Water Maze Test

Mice received visible platform pre-training on the first day, followed by hidden platform training for two days. In the hidden platform training, five sessions consisting of four trials were performed on two days. Mice were placed into the pool from four different directions in each of the four trials. The escape latency was measured. In the probe test, mice were allowed to swim for 60 sec. The number of crossings in each quadrant and the number of crossings over the platform area was analyzed by a video-tracking system (SMART, Panlab).

### Passive Avoidance Test

A chamber was divided into bright and dark compartments by a partition with a window. The floor of the dark compartment was composed of stainless steel rods connected to an electric shock generator. Mice were placed into the bright compartment, allowed to freely explore the chamber until they entered the dark compartment, and then were returned to the home cage. The third time the mice entered the dark compartment, they received an electric shock. On the second and third days, the time taken to enter the dark compartment was measured, up to a maximum of 5 min.

### Shuttle Avoidance Test

A chamber composed of two compartments, Compartments A and B, was divided by a partition with an openable gate. The floor of Compartment A was made of a stainless steel electric shock generator. Mice were placed in Compartment A with the gate closed, and allowed to explore for 5 min. A conditional stimulus (CS), 5 sec of tone, was given to the mice, while the gate was opened. The mice received the unconditional stimulus (US), a 5-sec electric shock, 10 sec after the CS (CS-US trial). The number of entries into Compartment B was counted during the CS presentation and 10 sec before the US. Each mouse received 20 CS-US trials each day, for two days. The number of successful responses, where the mice moved to Compartment B, was defined escape score.

### Fear Conditioning Test

Mice were habituated in a chamber for 2 min, followed by a 30-sec light stimulus (CS) paired with an electric shock during the last 3 sec of the CS (US). The mice were allowed to stay in the chamber for another 2 min. The CS-US pairing was repeated four times, and then the mice were returned to the home cage. The mice were placed back into the fear conditioning chamber 24 h later without the light cue, and the freezing time and the number of occurrences of tail-rattling were measured for 5 min (contextual conditioning). The mice were placed into a columnar chamber 1 hour later, and given a light stimulus (CS), and the freezing time was measured (cued conditioning). The assessment was performed by an observer blinded to the mouse genotype.

### Open Field Test

Mice were placed into the center of a circular open field, and allowed to explore for 30 min. The total distance moved and time spent in the center area were analyzed by a video-tracking system (SMART, Panlab).

### Dark/Light Transition Test

Mice were placed into a dark compartment of a two-compartment chamber, and were allowed to explore for 10 min. while being monitored by a video-tracking system (SMART, Panlab).

### Tail Suspension Test

The tails of mice were fastened to a bar, and then the mice were observed for 6 min. The first latency to immobility and immobility time were measured. The assessment was performed by an observer blinded to the mouse genotype.

### Statistical Analysis

Student’s *t* test or an ANOVA with a LSD post-hoc test was performed to evaluate the significance of the data.

## Supporting Information

Figure S1 **No difference in the reaction to the heat between α2AP^−/−^ and WT mice.** Mice were placed on a 55 or 58°C hot plate, and the first latency for them to lick their paws was measured. There was no significant difference in the reaction to the heat between α2AP^−/−^ and WT mice (WT and α2AP^−/−^ mice, n = 14 and 10, respectively). The values represent the means ±S.E. Significance was evaluated using Student’s *t*-test.
(TIF)

Figure S2 **The intact electric shock-induced acute freezing response in α2AP^−/−^ mice.** Mice were habituated in a different shape of chamber from the one used for the fear conditioning test for 2 min, followed by an electric shock during the last 3 sec. The freezing time was measured while the mice were allowed to stay in the chamber for another 2 min. This trial was repeated four times. There was no difference in the freezing time after each electric shock. (WT and α2AP^−/−^ mice, n = 14 and 10, respectively). Significance was evaluated using an ANOVA with a LSD post-hoc test.
(TIF)
